# Physicochemical features partially explain olfactory crossmodal correspondences

**DOI:** 10.1038/s41598-023-37770-1

**Published:** 2023-06-30

**Authors:** Ryan J. Ward, Sophie M. Wuerger, Maliha Ashraf, Alan Marshall

**Affiliations:** 1 School of Computer Science and Mathematics, Liverpool John Moores University, Liverpool, L3 3AF UK; 2 Digital Innovation Facility, University of Liverpool, Liverpool, L69 3RF UK; 3 Department of Electrical Engineering and Electronics, University of Liverpool, Liverpool, L69 3GJ UK; 4 Department of Psychology, University of Liverpool, Liverpool, L69 7ZA UK

**Keywords:** Biophysical chemistry, Analytical chemistry, Chemical biology, Biological physics, Electrical and electronic engineering, Biochemistry, Chemistry, Psychology, Human behaviour, Neuroscience, Olfactory system

## Abstract

During the olfactory perception process, our olfactory receptors are thought to recognize specific chemical features. These features may contribute towards explaining our crossmodal perception. The physicochemical features of odors can be extracted using an array of gas sensors, also known as an electronic nose. The present study investigates the role that the physicochemical features of olfactory stimuli play in explaining the nature and origin of olfactory crossmodal correspondences, which is a consistently overlooked aspect of prior work. Here, we answer the question of whether the physicochemical features of odors contribute towards explaining olfactory crossmodal correspondences and by how much. We found a similarity of 49% between the perceptual and the physicochemical spaces of our odors. All of our explored crossmodal correspondences namely, the angularity of shapes, smoothness of textures, perceived pleasantness, pitch, and colors have significant predictors for various physicochemical features, including aspects of intensity and odor quality. While it is generally recognized that olfactory perception is strongly shaped by context, experience, and learning, our findings show that a link, albeit small (6–23%), exists between olfactory crossmodal correspondences and their underlying physicochemical features.

## Introduction

Crossmodal correspondences are the consistent associations between stimulus features in different sensory modalities^1^. A large body of work has found consistent mappings between odors and different stimuli (i.e., the angularity of shapes^2^, fabric softness^3^, the smoothness of texture^4^, colors^5,6^, pitch^7^, and musical genres^8^). The underlying mechanisms behind crossmodal correspondences have a diverse characterization in the literature, with the most frequently concluded being hedonics^2,9–12^ with emotions^8,13^ being particularly important, semantics^1,5,8,11,14–16^, and natural co-occurrence^1,15,17,18^. These correspondences are also influenced by culture (i.e.,^19–21^). Olfactory perception occurs when volatile molecules enter the nasal cavity and are transduced by the olfactory receptors^22^. A neural signal is then transmitted to the olfactory system; at this stage, a neural representation of the perceptual and physical characteristics is formed^23^ and can be described semantically^24^ by many types of perceptual qualities (e.g., woody, floral, minty, etc.). The olfactory system also shares a common neural substrate with the limbic system, which plays a vital role in mood and emotional evaluation^25^, namely the amygdala. The bindings between odorous molecules and the olfactory receptors are thought to recognize specific chemical features^26^. For instance, odors with low molecular weight or low structural complexity or containing sulfur are often perceived to be unpleasant^24,27–29^. Humans possess thousands of olfactory receptors which enable us to finely discriminate a wide range of odors. Bushdid et al*.*^30^ controversially^31,32^ claim that humans are capable of discriminating up to a theoretical trillion odors. Some odors e.g. ethyl mercaptan can be detected as low as one part per billion^33^. The human sense of smell is remarkable for the detection of hazardous compounds^33^ but is flawed for identification even for commonly encountered odors in the absence of visual or contextual cues^34–37^. Odor naming can be difficult task due to the “tip of the nose” phenomenon^36^; however Jönsson et al*.*^35^ suggest that the “tip of the nose” phenomenon may not exist. They claim that this is not due to the commonly suggested weak connection between odors and their respective names, but rather a failure to know what the specific odor is. This f﻿l﻿awed identification of odors may also be attributed to ecological, cultural, or genetic factors; for example, languages with elaborate smell lexicons demonstrate improved odor identification (see^38^ for a review).

A notable part of human olfaction is hedonics; pleasantness plays a notable role in olfactory perception. All humans can say invariably about odors is whether they are pleasant or not^39^; this may also extend to edilbility^40,41^. When ordering a set of odorants based on the variance (principal component scores) of the odors physicochemical descriptors (i.e., fruity, floral, and aromatic), they also end up relatively ordered in terms of pleasantness^42^. This allowed Khan et al*.* to predict (r ≈ 0.5; *p* ≈ 0.004) the perceived pleasantness of > 50 molecules that were not part of their models^42^. Pleasantness has also emerged as a dominant dimension in the multidimensional analyses of perceptual odor spaces^42,43^. Most literature converges and suggests that pleasantness is a primary perceptual dimension of olfaction. Similarly, in olfactory crossmodal correspondences, a critical mediating factor is assumed to be hedonics^2,9–12^, and when considering semantic involvement, knowing what the odor is or not will increase the perceived pleasantness^44–47^. In other words, the primary dimension in both crossmodal correspondences and human olfaction is suggested to be hedonics and therefore implying a common denominator. Odor intensity is correlated with quality^48^ and hedonic strength^49^, suggesting a link between olfactory perception and the physicochemical aspects of odors.

There have been attempts to characterize a physicochemical odor space^28,42,50^; classically, molecules’ physical or chemical features often come from online datasets or specialized software and are coupled with semantic descriptors, such as “minty” or “sweet” and are usually provided to the participants for rating instead of being generated by the participants. The physical, chemical and/or molecular features have been linked to different aspects of human perception, namely the perceived pleasantness^42,51^, participant ratings of semantic descriptors^50^, similarity^52,53^, and “brightness”^54^. “Brightness” was once considered an amodal dimension shared across sensory experiences; however, today, it is considered a visual property. A long-standing suggestion is that the perceived brightness or intensity of the stimuli may be used to make crossmodal matches^1,54^. von Hornbostel believed that ”brightness” was a characteristic of all sensory modalities and suggested that these “brightness” judgments in the visual dimension were related to the molecular structure^54^.

The “chemical footprint” of odors can be transduced via an electronic nose (e-nose). Like the human olfactory system, an e-nose consists of an array of gas sensors, each with a limited detection capability, and relies on the quantity of sensors for specificity. This makes an e-nose a cost-effective and feasible solution for transducing the physicochemical features behind different gaseous compounds in the vapor phase^55^. E-noses can generally detect odors more efficiently than the human nose but only for odors which they were designed to detect. They are often fine-tuned to pick up on chemical properties of interest (i.e., essential oils^28,51,56–60^, TCA wine cork taint^61^, and even diabetes detection^62^). Sarno and Wijaya used a series of gas sensors to target carbon monoxide, carbon dioxide, acetone, and volatile organic compounds, which are biomarkers for diabetes in human breath^62^. E-noses have been linked to different aspects of human perception (i.e., perceived pleasentness^51,63^, perceived intensity^64^, color^58^, and perceptual descriptors^65^). The physicochemical properties of odors are a consistently overlooked aspect of prior work and are the primary motivation behind this research. We use the term physicochemical to refer to both the chemical (presence and quantity of specific gases) and physical (temperature, pressure, and humidity) features of our olfactory stimuli.

One of the major unresolved problems in olfaction research is the relation of olfactory perception to the physicochemical features of the stimuli. The present study was designed to investigate if the physicochemical features of odors are a contributory factor towards explaining olfactory crossmodal correspondences. Two different brands of olfactory stimuli were selected to give chemical diversity to the underlying physicochemical features, thereby demonstrating the robustness of their contribution. The stimuli set and the perceptual dimensions used were initially reported in our prior work^4^. Our first hypothesis is that a reasonable degree of similarity will be obtained between our olfactory stimuli in the physicochemical and perceptual space. Our second hypothesis is that the physicochemical features of odorous stimuli will contribute towards explaining the nature and origin of olfactory crossmodal correspondences. In our prior work^66^, we predicted the crossmodal correspondences of odors. Here, we build upon these findings by probing the relationship between the physicochemical features of odors and olfactory crossmodal correspondences and uncovering the extent of how much they contribute towards explaining olfactory crossmodal correspondences.

## Materials and methods

### Perceptual data

**Figure 1:**
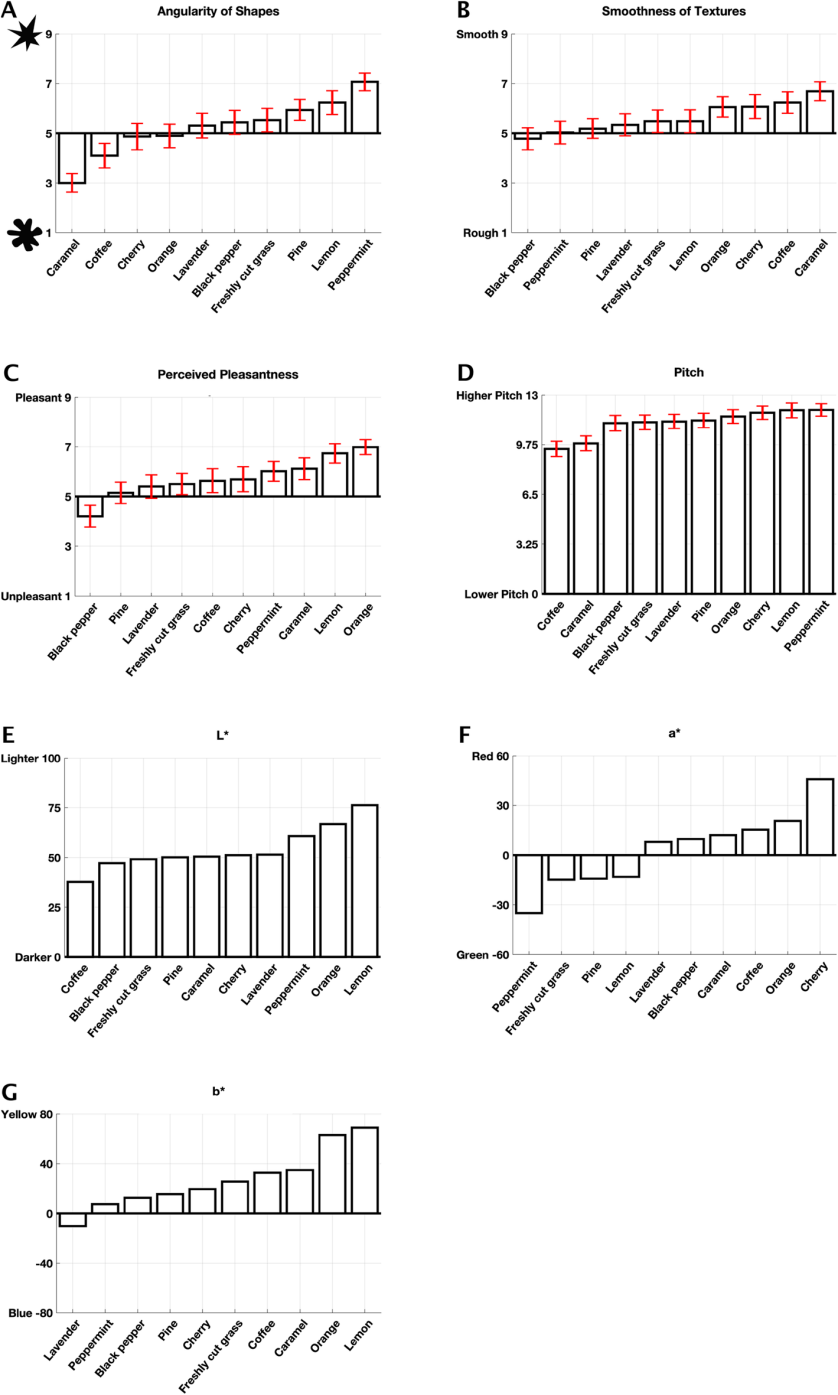
Underlying perceptual data. (**A**–**C**) Shows the mean scores for the angularity of shapes, smoothness of textures, and perceived pleasantness. (**D**) Shows ${log}_{2}$ of the mean pitch scores. (**E**–**G**) Shows the median values for the color dimensions L*, a*, and b*, respectively. Errors bars denote a 95% confidence interval.

We used the perceptual data collected in our prior work^4^, which explored an aggregate of olfactory crossmodal correspondences between olfactory stimuli and the angularity of shapes, smoothness of textures, perceived pleasantness, pitch, musical, and emotional dimensions. Five of the olfactory stimuli (black pepper, lavender, lemon, orange, and peppermint) were made by Miaroma™. The other five (caramel, cherry, coffee, freshly cut grass, and pine) were created by Mystic Moments™. The perceptual information gathered relates to the crossmodal correspondences between the odors and respective associations listed above. The results were obtained from 68 participants (45 females and 23 males, mean age of 26.75 years, standard deviation of 12.75 years) in a controlled environment (lightproof anechoic chamber equipped with an overhead luminaire (GLE-M5/32; GTI Graphic Technology Inc., Newburgh, NY)). The essential oils were placed in clear test tubes numbered 1 through 10 randomly and covered in white tape to avoid any associations between the color of the oil and potential crossmodal correspondences. The olfactory stimuli were presented to the participants in random order. Participants had to smell each odor at least once at the first instance of its involvement in the experiment; optionally, they could smell it again if they felt like they needed to. There was no limit imposed for the duration in which they could smell the essential oil. For more information about the underlying perceptual data including significance tests see^4^. The underlying perceptual data is shown in Fig. 1. Sections "Shape stimuli"–"Color stimuli" covers the specifics of how each perceptual dimension was collected.

#### Shape stimuli

**Figure 2:**
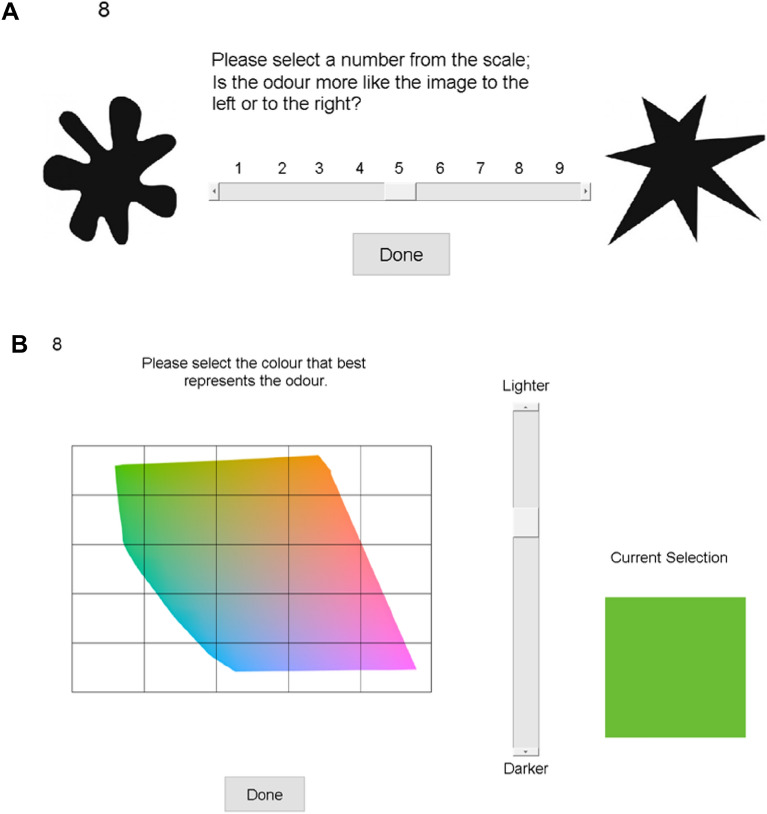
Scales used to collect olfactory-visual associations. (**A**) The scale that was used to rate the association of each odor with a round or angular shape. (**B**) Graphical user interface used to collect the color associated to each of the odors. The number in the top left refers to the odor to be presented to the participant.

A nine-point Likert scale was used with the images of “kiki” (angular shape) and “bouba” (rounded shape) images on the scales left and right sides (see^2^). The scale's midpoint was reserved for neutral (no opinion), see Fig. 2A.

#### Texture stimuli

A nine-point Likert scale was used with the words “rough” and “smooth” on the left and right-hand sides of the scale, respectively. Participants were presented with physical representative textures to aid them in their decision, with sandpaper representing “rough” and silk representing “smooth”. The participants had to feel the representative textures at least once during the question’s first appearance. The middle of the scale was reserved for neutral (no opinion). A Likert scale anchored with words was used for this task to align with prior literature^3^.

#### Pleasantness ratings

A nine-point Likert scale was used with the words “unpleasant” and “pleasant” on the left and right-hand sides of the scale, respectively. The midpoint of the scale was reserved for neutral (no opinion).

#### Pitch stimuli

A scale was constructed with the words “lower pitch” and “higher pitch” of the left and right-hand sides, respectively. The lower end of the scale was 20 Hz, the higher end was 20 kHz. Each time the slider was adjusted, a sinusoidal tone lasting 1 s in length was presented to the participant at the specified frequency, indicated by the slider’s position expressed in a linear fashion. To reduce the number of potential selections, participants were played a sample from each end of the scale followed by a sample halfway between the two. The lower end was incrementally increased, and the higher end was incrementally decreased until the participants could hear the tone. The participants responded with higher or lower, and the respective sample was played between the last played sample and the previous played upper or lower bound. When the participant felt like the pitch matched the odor, the last played frequency was saved. The experimenter adjusted the slider for this task the upper and lower positions were played only for the first odor or if the participant felt like they needed to hear it again.

#### Color stimuli

All visual stimuli, including the color stimuli, were presented on a calibrated EIZO ColorEdge CG243W monitor. Participants could slide through the L*a*b* color space by adjusting the L* (perceptual lightness) value, all a* (red-green) and b*(blue-yellow) colors that fit into the sRGB color gamut were displayed for the currently selected L* value. Participants could freely choose a color from the current slice; once the color was selected, a sample color patch was displayed in the corner, see Fig. 2B. The participant’s final color selection was saved.

### Electronic nose

An electronic nose (e-nose) was used for extracting a subset the physicochemical features from the odors. An e-nose was chosen as it is low-cost, portable, and capable of transducing the physicochemical features of the odors. An e-nose uses an array of semi-selective gas sensors to determine the underlying physicochemical characteristics^67^. The e-nose initially presented in^56^ and modified in^58^ was used to record the odors. The sensors used in the e-nose, along with their specifications, are shown in Table 1. The e-nose was developed at the University of Liverpool’s Immersive Reality Laboratory, see^58^ for more information about the operating principles of the e-nose. The e-nose handles numerous gases using several sensors by utilizing cross-reactivity, where gaseous mixtures (essential oils) will produce variable responses across the sensor array. The differentiation of gases is determined by the amplitude of the sensor responses. The caveats of using an e-nose would be that they are usually designed to detect specific compounds of interest meaning there could be potentially important compounds that are not being detected and therefore analyzed. Another disadvantage in our case is that some of the values obtained from the e-nose are qualitative and not quantitative. See^68^ for the strengths and weaknesses of using e-noses. An e-nose would only capture aspects relating to the physical and chemical features of given odors. Linking features from an e-nose to human perception would require a relationship to exist between the physicochemical features of the odor and the odors corresponding perception. The linking would also require an accompanying pattern recognition system^55^ to recognize regularities and patterns in the data. In Table 1 “Gas” represents total volatile organic compounds, “Air Quality” is a measure of a series of harmful gases, “Temperature” refers to the temperature in °C, “Pressure” refers to barometric pressure and is captured in kPa, and “Humidity” is captured as a relative % (0–100%).

**Table 1:** Sensors used in the e-nose with their range (ppm) and detectable gases.

Sensor name	Detection range (ppm)	Subset of detectable substances	Sensor output name
MP503	10–1000	Carbon monoxide, alcohol, acetone, HCHO (formaldehyde)	Air quality
BME680	0–500	Volatile organic compounds	Temperature, humidity, pressure and gas
MQ3	0.05–10	Alcohol, benzine, methane, hexane, liquefied petroleum gases and carbon monoxide	MQ3
MQ5	200–10,000	Liquefied petroleum gases, natural gas, town gas, alcohol and smoke	MQ5
MQ9	10–1000 CO 100–10,000 Gas	Carbon Monoxide, Coal Gas & Liquefied Gas	MQ9
WSP2110	1–50	HCHO (formaldehyde), toluene, methanol, benzene and alcohol	HCHO

It is important to note that the sensors may respond to gases not included in this table.

### Odor recordings

Odors were prepared and presented to the e-nose in the same manner as presented to the participants in our prior work^4^. This was done because consistent crossmodal correspondences can occur between odors because of intensity^2,18,46^, and temperature^21,69^. We wanted to collect physicochemical features in a manner that best represented the stimuli as they were initially presented to the participants. Odors were placed in the e-nose, sealed inside, and left recording for 10 min. This was repeated ten times for each of the ten odors (black pepper, caramel, cherry, coffee, freshly cut grass, lavender, lemon, orange, peppermint, and pine); a total of 100 recordings were prepared for the experiments. As the odor recordings were in a time series format, the recordings were pre-processed before the analysis to reduce dimensionality and remove some sensor noise from the underlying signals. First, the mean was calculated for each sensor over 1-s intervals, resulting in a 600 × 9 matrix; each sensor’s signal was then smoothed by applying a three-point centered moving average filter. Each column in this matrix is the physicochemical features (air quality, temperature, pressure, humidity, gas, MQ3, MQ5, MQ9, and HCHO), and the rows correspond to a time point between 1 and 600 s. After filtering, each sensor's median value was added to the dataset, generating a 100 × 9 dataset for the physicochemical features. Each feature's mean value was then calculated independently for each odor, resulting in a 10 × 9 dataset; these are the physicochemical values used in all statistical analyses. Each row in this matrix corresponds to an odor, and each column is a different physicochemical feature. The mean physicochemical features were then repeated sixty-eight times (the number of participants) so that the physicochemical features could be aligned with the perceptual responses.

### Statistical analyses

The analysis was performed using the Statistics Toolbox of MATLAB R2018b.

### Ethics statement

This study was conducted in accordance with the Declaration of Helsinki and had ethical approval from the Department of Psychology at the University of Liverpool. The ethics committee / institutional Review Board of the Department of Psychology at the University of Liverpool approved all experimental protocols. All participants provided informed written consent, and a fraction of the participants received course credit in exchange for their participation.

## Results

### Exploratory analysis

**Figure 3:**
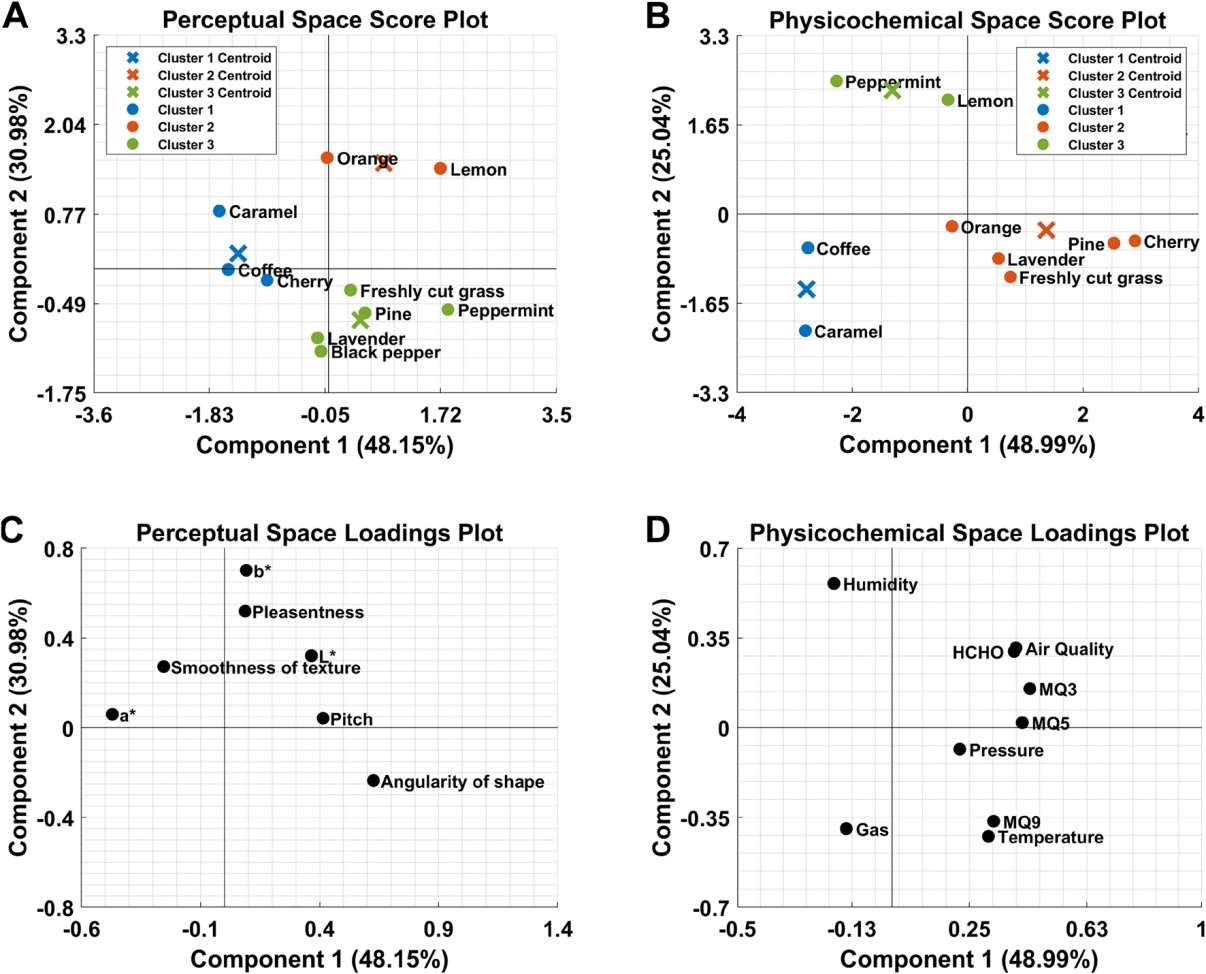
Principal components score plots. Principal components in (**A**) perceptual space, (**B**) physicochemical space and their loadings in (**C**), and (**D**) respectively. Both score plots are based on the PCA correlation matrix.

Two datasets were constructed to test our hypotheses, one for the perceptual and the other for the physicochemical. To construct the perceptual dataset, the colors were first converted to the L*a*b* color space^70^ (chosen for its approximate perceptual uniformity). They were then coupled with the raw values for the angularity of shapes, smoothness of textures, perceived pleasantness, and pitch ratings. The physicochemical dataset consists of the mean physicochemical features collected by the e-nose (air quality, temperature, humidity, pressure, gas, MQ3, MQ5, MQ9, and HCHO) for each odor. Principal component analysis (PCA) was first conducted on the mean perceptual and physicochemical data to visualize the interrelationship between the odors in the two spaces. To prepare the datasets for PCA, both the pitch ratings and color dimensions were rescaled between 1 and 9. Z-score normalization was then conducted on both datasets separately; the population standard deviation and mean of all the dataset was used for the perceptual dataset. For the physicochemical dataset, the z-score normalization used the population standard deviation and the mean of the columns. The distance between two points indicates how similar two odors are in their respective space, with the closer points indicating a higher degree of similarity. Based on inspection of the scree plots, the first two components for both the perceptual and physicochemical data were kept; no rotations were performed as we wanted to keep the two spaces as comparable as possible. The perceptual dataset’s first two components explain 79.13% of the total variance, 48.15%, and 30.98%, respectively, as shown in Fig. 3A. The first two components for the physicochemical dataset explain 74.03% of the total variance, 48.99%, and 25.04%, respectively, as shown in Fig. 3B. For the perceptual space we can see that that the angularity of shapes explains the most variation on the x axis and the b* color dimension explains the most variation on the y axis (see Fig. 3C). For the physicochemical space we can see that the MQ3 response explains most variation on the x axis with humidity explaining the most on the y axis (see Fig. 3D).

To determine the groups that archived similar perceptual/physicochemical scores, k-mean cluster analysis was conducted on all the principal component scores. This process produces clusters based on feature similarity, where each cluster contains similar principal component scores across all dimensions. From Fig. 3A, we can see that (caramel, coffee, and cherry), (orange and lemon), and (freshly cut grass, lavender, pine, peppermint, and black pepper) obtained comparable scores making them perceptually similar. From Fig. 3B, we can see that (coffee and caramel), (peppermint and lemon), and (black pepper, lavender, freshly cut grass, orange, pine, and cherry) obtained comparable scores, making the odors in the vapor phase similar both physically and chemically. Comparing the cluster groupings from Fig. 3A with Fig. 3B, we can see moderate overlap in the physicochemical and perceptual spaces with (coffee and caramel), and (lavender, freshly cut grass, pine, and black pepper) are in the same cluster in both cases. Therefore, showing a moderate overlap between odors in the perceptual and physicochemical spaces implying that a reasonable degree of similarity exists between these two spaces. To analyze the true extent of this overlap, we decided to continue with a Procrustes analysis^71,72^.

### Procrustes analysis

**Figure 4:**
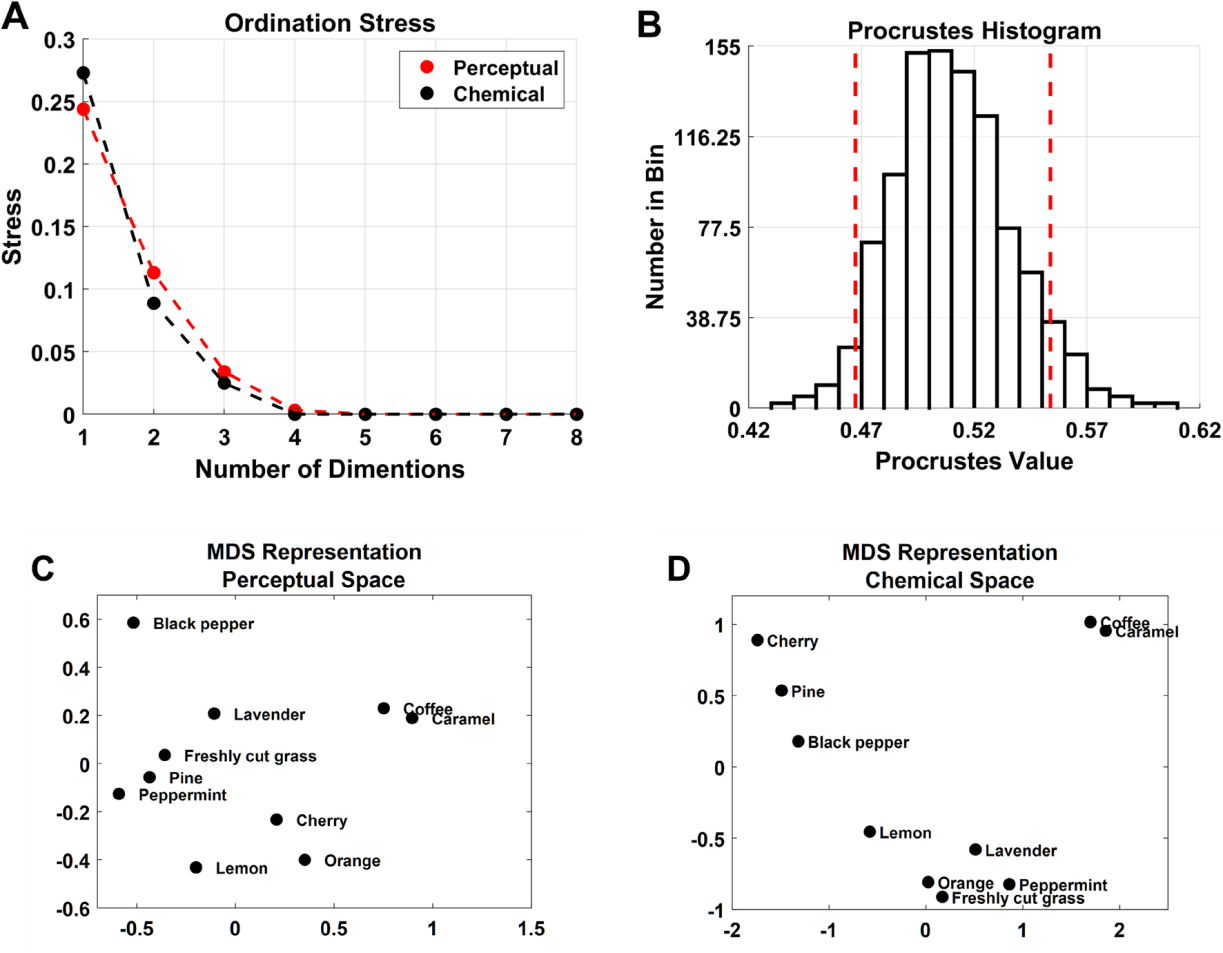
Procrustes analysis plots and multidimensional scaling representations. (**A**) Ordination stress scree plot. (**B**) Histogram plot of 1000 generated Procrustes values using a random location with uncorrelated coordinates and from a scaled *p*-dimensional normal distribution. The vertical dashed lines indicate a 95% confidence interval. (**C**) Example MDS plot of the perceptual space. (**D)** Example MDS plot of the physicochemical space. The shape of the MDS plots will vary based on the initial random start location.

A Procrustes analysis was performed to quantify how much overlap there is between the odors in the physicochemical and perceptual space. That is, how well do these two spaces fit together by transforming the shapes of the physicochemical and perceptual spaces to archive a maximal superimposition. The Procrustes analysis is a rigid shape analysis that finds the “best” fit between two or more multidimensional shapes using isomorphic scaling, rotation, and translation. The physicochemical and perceptual space was fitted to the physical space via the Procrustes function in MATLAB^71^. The algorithm fits the points between the perceptual and physicochemical space using the best shape-preserving Euclidean transformations matching the physicochemical and perceptual spaces to the physical space. As multidimensional scaling (MDS) provides relative points in space, a Procrustes analysis can be performed on the resulting matrix^71,72^ with the goodness of fit criterion defined as the sum of squared errors. The output consists of the distance of points in space where lesser values indicate a better fit, with zero being a perfect fit and one being entirely dissimilar. The dataset was pre-processed in the same manner as performed in the exploratory factor analysis above. A scree plot showing ordination stress was constructed to determine how many dimensions are required to explain the data sufficiently (Fig. 4A). This revealed that the first two dimensions are sufficient for visualization for both the physicochemical and perceptual dimensions. Therefore, we decided to plot the MDS maps in two dimensions. Looking at both the PCA scores plots (Fig. 3A,B) and the MDS maps (Fig. 4C,D), we can see that the perceptual and physicochemical spaces are visually similar. One thousand simulations from a random location with uncorrelated coordinates and an appropriately scaled *p*-dimensional normal distribution were run using the first three dimensions to determine how similar these two spaces are. The goodness of fit was calculated for each pair of MDS solutions (mean = 0.51, min = 0.43, max = 0.60, see Fig. 4B), where a value of 0 indicates a perfect alignment. This revealed a 49% (1–0.51 * 100) similarity on average between the physicochemical and perceptual spaces in the physical space, meaning that perceptually similar odors are also similar in the physicochemical space to 49%.

### Canonical correlation analysis

**Figure 5:**
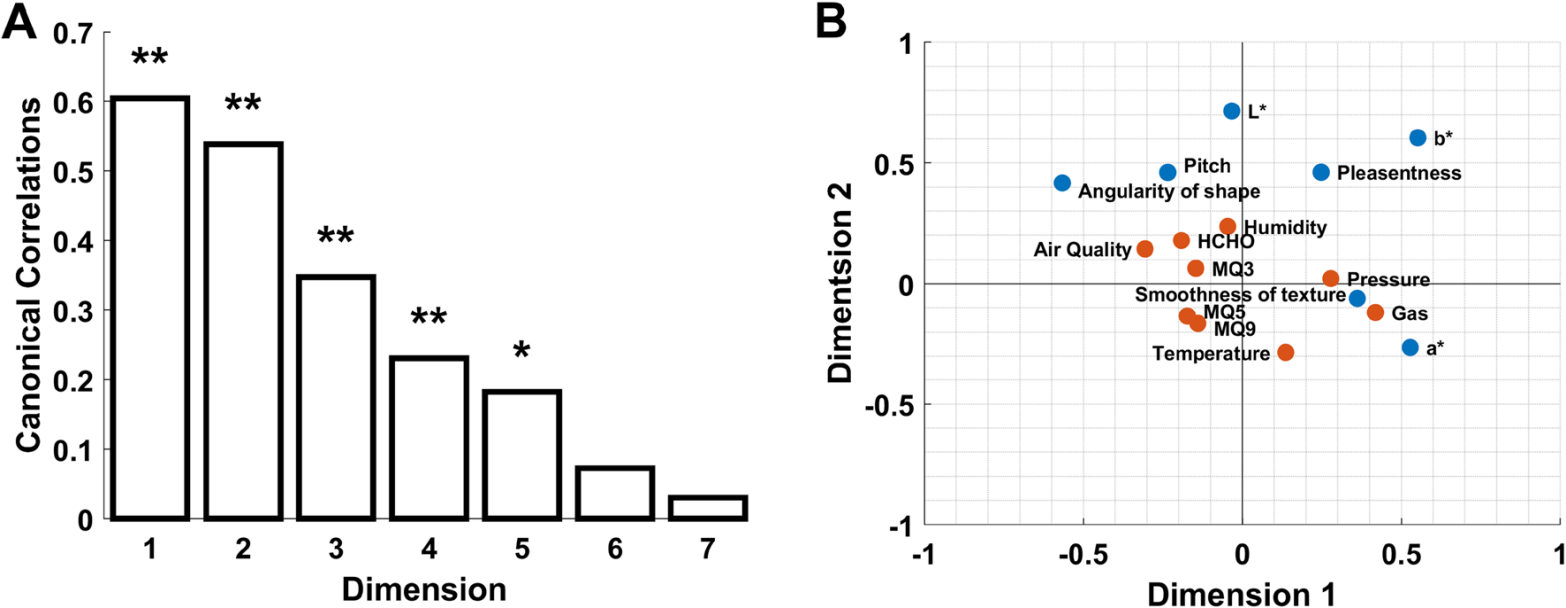
Canonical correlation results. (**A**) Canonical correlations for the seven canonical variable pairs. ** denote *p* < 0.0001, * denotes *p* < 0.05. (**B**) Canonical correlation loadings plot showing the first two dimensions.

A canonical correlation analysis was conducted to explore the relationship between the perceptual and physicochemical features. The analysis produced seven functions, with the first five being statistically significant, and have Wilks’s lambdas of 0.36 (*p* < 0.0001), 0.57 (*p* < 0.0001), 0.80 (*p* < 0.0001), 0.91 (*p* < 0.0001), and 0.96 (*p* < 0.05), respectively. The canonical correlations for the functions are 0.60, 0.54, 0.35, 0.23, and 0.18, as shown in Fig. 5A. Based on inspection of Fig. 5A, we decided that the first two dimensions were sufficient as their correlation coefficient were greater than the cutoff value of 0.45 which has been considered as “fair”^73^. Figure 5B shows the loadings of the first two dimensions.

From Fig. 5B we can see that the physicochemical features contribute towards the perceptual dimensions. The “Air Quality” sensor detects a wide range of harmful gases for indoor air conditions. The “Gas” feature represents air quality that is mainly affected by volatile organic compounds. Based on the loadings of either “Air Quality” and/or “Gas” on the angularity of shape, pitch, L*, smoothness of texture, and a*, it could be concluded that the quality of the stimuli has an influence on crossmodal correspondences. The loading of “HCHO” on the same perceptual dimensions suggests that the chemical compound formaldehyde may be responsible. Temperature, humidity, and pressure will interact in complex ways to determine the intensity of the odor. It is important to note it is not possible to rule out the involvement of other physicochemical features in contributing to intensity (i.e., the concentration of specific chemicals). This manuscript will focus on the physical temperature, humidity, and pressure as aspects of intensity, as these three features would be a constant underlying all odors. Based on the loadings of either “Temperature”, “Pressure”, and/or “Humidity” onto all of the perceptual dimensions, albeit weakly in some cases, suggests that intensity also plays a contributory role in explaining the nature and origin of crossmodal correspondences. However, the lack of loadings on the pleasantness and b* dimension suggests that they are more robust against the physicochemical features contribution. The physicochemical features provided by the e-nose are a composite of the entire smell; therefore, it would be ideal to see how the physicochemical features impact olfactory crossmodal correspondences as a whole instead of its individual elements; consequently, we proceeded to create generalized linear mixed-effects models.

### Generalized linear mixed-effects model results

The generalized linear mixed-effects models (GLME) were created using the raw perceptual ratings as the dependent variable and the mean physicochemical features for each odor as the independent. The physicochemical features used in this analysis are air quality, temperature, pressure, humidity, gas, MQ3, MQ5, MQ9, and HCHO. Each model was only fitted to one perceptual dimension at a time resulting in seven different models. First, multicollinearity was tested in the physicochemical dataset. This revealed multicollinearity between Air Quality (VIF = 16.8302) and MQ3 (VIF = 17.0148); therefore, we decided to remove the MQ3 feature from the dataset. The VIF values were rechecked after removing the MQ3 feature revealing no multicollinearity (all VIF values are less than 5) in the physicochemical dataset. We then proceeded to fit the GLME’s (Bonferroni corrected for the number of fixed effects coefficients (α = 0.0062)), treating the participants and the odors as a random factor. Coefficients with a *p*-value greater than our Bonferroni corrected alpha were not included in Table 2. Full details for each model can be found in the supplementary materials (Table S1).

**Table 2:** GLME results for all perceptual dimensions.

Model	Model fit statistics (Akaike information criterion (AIC), Conditional *R*^2^ (C-*R*^2^), Marginal *R*^2^ (M-*R*^2^))			Coefficients name (estimate, SE, t-stat, *p*-value)
	AIC	C-*R*^2^	M-*R*^2^	
Angularity of shape	3118.9	0.18	0.18	Temperature (− 1.30, 0.22, − 5.87, *p* < 0.0001)
Smoothness of texture	2986.5	0.10	0.06	Gas (0.65, 0.22, 2.88, *p* = 0.0040)
Perceived pleasantness	2953.4	0.20	0.10	Gas (1.21, 0.21, 5.67, *p* < 0.0001) MQ5 (1.37, 0.22, 6.29, *p* < 0.0001) HCHO (1.07, 0.21, 5.19, *p* < 0 .0001)
Pitch	11,906	0.36	0.09	HCHO (1989.6, 485.74, 4.09, *p* < 0.0001)
L*	5931	0.25	0.16	Air Quality (8.43, 1.99, 4.23, *p* < 0.0001) Pressure (− 6.83, 1.31, − 5.21, *p* < 0.0001) Gas (11.36, 1.91, 5.95, *p* < 0.0001) MQ5 (− 16.60, 1.94, − 8.57, *p* < 0.0001) MQ9 (7.03, 2.16, 3.26, *p* = 0.0012) HCHO (13.13, 1.84, 7.14, *p* < 0.0001)
a*	6521.2	0.22	0.17	Temperature (12.09, 2.76, 4.39, *p* < 0.0001) Humidity (14.79, 3.61, 4.10, *p* < 0.0001) Gas (10.23, 2.99, 3.42, *p* = 0.0007)
b*	6703.4	0.25	0.23	Air Quality (25.87, 3.61, 7.18, *p* < 0.0001) Temperature (14.70, 3.18, 4.62, *p* < 0.0001) Gas (21.63, 3.46, 6.26, *p* < 0.0001) MQ5 (− 38.48, 3.50, − 10.98, *p* < 0.0001) MQ9 (− 19.69, 3.90, − 5.05, *p* < 0.0001) HCHO (15.28, 3.33, 4.59, *p* < 0.0001)

The degrees of freedom for all coefficients in the table is 671 with the exception of the pitch model, where the degrees of freedom is 591. Marginal *R*^2^ is the variance explained by the fixed factors, and conditional *R*^2^ is the variance explained by the entire model^74^. The Wilkinson model formula that was used is: Perceptual dimension ~ 1 + Air Quality + Temperature + Pressure + Humidity + Gas + MQ5 + MQ9 + HCHO + (1 | Participant ID) + (1 | Odor ID).

The results from Table 2 confirm the findings from the canonical correlation analysis and tell us that physicochemical features are a contributory factor towards explaining people’s crossmodal correspondences. That is, when treating the participants and the olfactory stimuli as a random effect, significant coefficients were found in all of the generated models. These models also show that between 6–23% variance is explained by the fixed effects (physicochemical features). Specifically, it shows that the perceptual dimension for the angularity of shape is affected by an aspect of intensity (temperature). The smoothness of texture, perceived pleasantness, and pitch are affected by odor quality (gas and/or HCHO), and the color dimensions (L*, a*, and b*) are affected by aspects of odor quality (air quality, gas, and/or HCHO) and intensity (temperature, pressure, and/or humidity).

## Discussion

The results from the present study introduced the concept that the physicochemical features of odors play a role in explaining olfactory crossmodal correspondences, which has been a consistently overlooked aspect in prior work. Other influential factors towards explaining the nature and origin of olfactory crossmodal correspondences include hedonics^2,9–12^, semantics^1,5,8,11,14–16^, and natural co-occurrence^1,15,17,18^. We use the term physicochemical features to refer to our olfactory stimuli' (essential oils) physical and chemical characteristics. Here we show that the underlying attributes of odorous stimuli contribute to the crossmodal correspondences between odors and the angularity of shapes, smoothness of textures, perceived pleasantness, pitch, and color dimensions (L*, a*, and b*). Olfactory perception is modulated or influenced by several factors, such as expectations^75^, context^76^, multisensory convergence^77^, in utero neuroanatomical development^78^, and is a heavily learned process^79^. It is important to note that the dominant aspects of olfactory perception, such as context, will not be reflected in the physicochemical features^42,51^. Our findings are in alignment with the concept that hedonic perception is a very influential factor in olfactory perception. We found significant predictors from aspects of intensity and/or odor quality in all of our explored modalities (the angularity of shape, the smoothness of texture, perceived pleasantness, pitch, and colors). Moreover, recent lines of research suggest that hedonic perception is partly innate^29,42,51,80,81^, which is in contrast to the popular view that hedonic aspects are only shaped by experience. Nevertheless, the link between the physicochemical features and this partially innate and hardwired link of percept remains elusive and suggestive. This could be because olfactory stimuli do not vary continuously in stimulus space, and the dimensionality of the olfactory perceptual space is unknown^43,50,82^. The human olfactory system has multiple levels of plasticity^83,84^ that reflect a beneficial evolutionary mechanism to reject hazardous compounds. For instance, the odor ethyl mercaptan is often added to propane as a warning agent^33,39^. Rats bred for several generations are averse to the smell of predators even if raised in a predator-free enviroment^85^. The hedonic perception of odors is a complex process that involves learned and controversially innate components (see^39^ for further discussion).

Our first hypothesis was that there is a reasonable degree of similarity between our olfactory stimuli in the physicochemical and our crossmodal perceptual space. A Procrustes analysis with 1000 simulations was conducted on our physicochemical and perceptual spaces in the physical space revealing an average similarity rating of 49%. The finding that the physicochemical and perceptual spaces are similar corroborates the findings of Koulakov et al*.,* where a smooth curved surface with a small dimensionality can approximate the responses of human observers for monomolecular odors. Snitz et al*.* proposed a quantitative and robust method for measuring intricacy that depends on more intricate stimuli evoking a larger variance in the perceptual responses of participants^86^. The notion of intricacy and complexity of the stimuli could be embedded in both the physicochemical features^87^ and the perceptual ratings^86^ provided by the participants and is another plausible reason for explaining the findings reported in this study. Less chemically complex odors would have produced a simpler response in the electronic nose^88^. Comparatively, if less intricate stimuli produced less variance in the perceptual data, this may be a means of mapping the olfactory stimuli from one space to another. It is important to note that involvement from other mechanisms cannot be ruled out; for example, results from our GLMEs revealed that there could be contributions from the quality of the olfactory stimuli and intensity. Moreover, it has been demonstrated that the characteristic response patterns provided by an electronic nose can encapsulate aspects of perceived intensity^64,89^, which could also be reflected in the participant's crossmodal ratings. For instance, more intense odors are associated with darker colors^90,91^ and more angular shapes^2,5^. Our findings can, at least in part, corroborate these claims, as aspects of intensity ("Temperature” or “Pressure”) is a significant predictor with our perceptual dimensions for the angularity of shapes and the lightness of color (L*).

Our second hypothesis was that odorous stimuli' physicochemical features would contribute to explaining the nature and origin of olfactory crossmodal correspondences. GLMEs were created using the raw perceptual data and the mean physicochemical features revealing that the models captured 10–36% (conditional *R*^2^) of the total variance and between 6–23% (marginal R^2^) of variance is explained by the fixed effects, therefore, supporting our second hypothesis. Spence stated that crossmodal correspondences might occur at a low-level (amodal stimulus properties, such as duration) and at a high-level (semantics and hedonics)^1^. Here we claim that the physicochemical features are also a contributory factor, albeit weakly but contributory non the less. In our prior work^58,66^, we found that crossmodal correspondences were predictable using the underlying physicochemical features, consequently presenting a suggestive relationship between the two. Here we confirmed this relationship and uncovered the degree in which the physicochemical features contribute towards explaining olfactory crossmodal correspondences (6–23% dependent on the respective crossmodal correspondence); which is the novel contribution of the work conducted in this manuscript. Recent studies support a relationship between hedonic perception and their physicochemical features or molecular structure^39,42,51,63,80^. This link between the physicochemical features and hedonic perception may be attributed to various factors including but potentially not limited to—intensity^50,64,92^, complexity or intricacy^10,29,86,93^, and odor quality^7,94–96^. The perceived intensity of the stimuli can be expressed as a logarithmic function of stimuli concentration^97^. In the case of hedonic mediation, a few studies have linked the molecular properties^29,42^ and the physicochemical features^51,63^ of odors to their perceived pleasantness. The work presented in Khan et al*.* provided a new view that the pleasantness of unfamiliar and monomolecular odors could be partly explained by the physicochemical properties of the odors molecules^42^, indicating that olfaction may not be as subjective as previously believed.

Hanson-Vaux et al*.*’s results suggest that more intense odors were associated with a more angular shape^2^. However, it is important to note that the different physical characteristics of odors will interact in complex ways to determine the intensity of odor. For example, consider the interaction between temperature, humidity, and pressure. The GLME for the angularity of shapes captured “Temperature” as a significant predictor indicating that the intensity of the odor could have played a role in the angularity of shapes associations.

Most of the work relating the physicochemical^51,63^ or molecular features^29,42^ to human perception has focused on perceived pleasantness. Haddad et al*.* showed it was possible to link the physicochemical features provided by an electronic nose to the perceived pleasantness using an artificial neural network with a singular hidden layer and five neurons^51^. They suggested that their findings were attributed to a partly hard-wired and innate link in olfactory perception and not due to intensity. Khan et al*.* linked descriptors provided by Dravnieks’ *Atlas of Odor Character Profiles*, where ≈ 150 olfactory experts ranked 160 odors against 146 verbal descriptors^42^. They found that when ordering physicochemical properties based on their variance (principal component scores), they also get roughly ordered by perceptual pleasantness. This, in turn, allowed them to predict the pleasantness of molecules similar to the work presented by Haddad et al*.* they also claim that olfactory pleasantness is partly innate^42^. We also found that the perceived pleasantness can be linked to the physicochemical features of the odors. Our pleasantness model captured significant predictors on the “Gas,” “MQ5,” and “HCHO” components indicating that a relationship exists between the physicochemical features and their perceived pleasantness, and in turn, suggesting they can be predicted, which corroborates the findings of our prior work^66^. However, our perceived pleasantness model captured the second lowest amount of explained variance captured by the whole model, indicating that in terms of prediction, it may be one of the most challenging dimensions to model in comparison to our other olfactory crossmodal correspondences (i.e., auditory pitch).

Olfactory-pitch correspondences have been shown to be affected by the quality of the odor rather than pleasantness or intensity^7,10^. For instance, Belkin et al*.*’s results suggest that odor-pitch correspondences were matched based on perpetual features as opposed to perceived pleasantness and intensity^7^. Crisinel and Spence's results suggest that their odor-pitch correspondences are influenced by both pleasantness and complexity, but not intensity^10^. Essential oils can create secondary air pollutants which are caused by a reaction to the air that affect air quality. Air quality is one of the factors that explain the nature and origin of odor-pitch correspondences^7,10^. It has also been shown that essential oils can have a negative impact on air quality due to these secondary organic aerosols (see^98^ for a review). Formaldehyde (HCHO) is one of the secondary organic aerosols that can be created^99^ and was also transduced by our e-nose. The finding that “HCHO” is a significant predictor for pitch further supports that odor quality is a contributory factor for odor-pitch correspondences. Temperature-based crossmodal correspondences have also been documented, namely between color and pitch (see^69^ for a review on temperature-based crossmodal correspondences). Wang and Spence demonstrated the existence of crossmodal correspondence between pitch, tempo, and temperature (imagined or physically present)^100^. They found that an imagined cold drink was associated with a higher pitch soundtrack and a significantly faster tempo. They also found similar results with physically hot, room temperature, and cold drinks. However, here we did not find that temperature is a significant predictor for pitch, which could suggest a more perceptual relationship rather than a physical.

There is no shortage of literature documenting consistent olfactory-color correspondences (see^6^ for a review). However, the dominating aspect that has arisen from the literature relates to the familiarity and identifiability (semantics) of the odors^4,6^. Emotional mediation can also play a part in the case of unfamiliar odors, such as perfumes^13^. Gilbert et al*.* showed that color selections differed significantly as a function of emotion and indicated that the expectations of color for beverages could be elicited based solely on a verbal descriptor^101^. Although we could not directly compare if the color selections in the L*a*b* color space are more dependent on the physicochemical features or emotions, it would be an exciting topic for future work. Kemp et al*.* investigated the effects of perceived intensity on color correspondences^90^. They have shown that the stronger the odor’s perceived intensity, the darker the color associated with it. Although we cannot conclude the directionality (more or less intense) with our data, “Pressure” was found to be a significant predictor for the L* (lightness of color) channel indicating involvement from intensity.

The hue-heat hypothesis states that color influences temperature perception^102^. However, the inverse effect is noticed here where the physicochemical property “Temperature” was a significant predictor for the a* and b* color channels models but not the L* channel suggesting that the physical temperature of the odor affects the hue associations but not how light the color association is. It is worth noting that the current literature describing crossmodal correspondences has portrayed these associations as bidirectional; the finding that “Temperature” is a significant predictor with both a* and b* models provides further evidence of this. A patent developed at Lorraine University, France (patent FR no. 1255688) uses an artificial neural network to generate chromatic cards using the chemical composition and a library of sensory descriptors. Our findings can corroborate this and suggest that olfactory-color correspondences can still be predicted, at least in part, without the need for sensory descriptors. Jacquot et al*.* used these chromatic cards to convey the odors of cucumber, lavender, and peppermint^91^. British and French participants were shown these cards and asked to associate one of these three odors. The results revealed that the cards evoked the appropriate odor in both populations. Ho et al*.* demonstrated that color (red vs. blue) could modulate temperature judgments. Their findings are in contrast with the popular notion that red colors are warm and blue colors are cold and show the opposite. In the L*a*b* color space, L* is the lightness value where a decrease in this value would result in a darker color, a*axis is green–red, and the b* represents blue–yellow. “Temperature” in our case is a significant predictor in our a* and b* models but not our L* model. Ward et al*.* demonstrated that the physicochemical features could predict the crossmodal correspondences of odors including odor-color correspondences^58,66^. Here we provide supporting evidence for this claim as between 10–36% of the variance was explained by the whole models for our perceptual dimensions.

Michael et al*.* state that visual cues may dominate and guide temperature-related responses^103^. Michael et al*.* go on to explain that this may be attributed to lateralized patterns. That is, red-warming associations are more frequently reported after stimulation to the left nostril, and green-cooling associations are more frequently reported after stimulation to the right^104^. However, this lateralized pattern is only present when the olfactory stimuli are coupled with colors^103,104^. The essential oils, in our case, were wrapped in white tape to avoid any undesirable bias induced by the color of the oil. The participants also were not asked to rate the temperature of the stimuli, yet the absolute temperature of the stimuli is a significant predictor in our models for the angularity of shapes, a*, and b*color dimensions. One plausible explanation for this and indeed all temperature-based findings reported in this study is that temperature is a key factor towards the intensity of the odor. One of the issues researchers are working on is whether temperature-based correspondences are innate or acquired through experience^69^.

Although these findings help to explore the link between the physicochemical features of odors and crossmodal correspondences, the extent still remains to be investigated. In particular, a larger sample size of odors, including novel odorants and more crossmodal correspondences would need to be explored to determine the true extent that the physicochemical features contribute to our crossmodal perception. A 49% overlap was observed between the physicochemical and perceptual spaces in the physical space. However, the similarity between these two spaces will most likely increase with novel odors. In other words, the less familiarity the odor has in the general population, the greater the variation that the physicochemical features can theoretically explain. This should allow for more variation to be captured with predictive models. An important caveat of our findings is the relatively simple nature of our predictive model. Although a GLME helps simplify a complex problem and accounted for between 10–36% (conditional *R*^2^) of the explained variance of the whole models. More advanced prediction algorithms could better capture the relationship in the underlying data, for example, Gaussian Process Regression^58,66^ or an Artificial Neural Network^63^. In our prior work^66^, we predicted the crossmodal correspondences of odors. Here we show that this link is still there when the underlying data was not processed to maximize the predictive capabilities. We also quantified the relationship between the physicochemical features of odors and their respective crossmodal correspondences while uncovering which physicochemical features contribute towards olfactory crossmodal correspondences (see Table 2). Finally, here we delve into the possible reasons why this link may exist. Future work could include investigating the extent that crossmodal interactions can be predicted (i.e., can odor-temperature correspondences be predicted using the underlying physicochemical features?). This would involve training and testing a regression model, such as Gaussian Process Regression using a leave one odor out approach to determine if crossmodal correspondences can still be predicted even if the odors are unseen to the generated models (see ^58,66^). The role that the physicochemical features play in explaining olfactory crossmodal correspondences could be explored further by characterizing the olfactory stimuli directly before or after presenting them to the participant; therefore, including environmental parameters (i.e., room temperature) in the generated signals. Moreover, the role of physicochemical features in explaining other crossmodal interactions could also be explored; this could include musical notes^10^, tempo^100^, and the perceived masculinity/femininity^18^ of odors.

## Conclusion

The nature and origin of olfactory crossmodal correspondences have largely been characterized as originating from hedonics, semantics, and natural co-occurrence. Here, we found a small link between the physicochemical features of odors and crossmodal correspondences, with 6–23% of variance being explained by the physicochemical features. This link may be attributed to intensity, odor quality, and/or the complexity/intricacy of the stimuli which could be embedded in the underlying physicochemical features transduced by our electronic nose and expressed in the collected perceptual ratings. Overall, our results show that the physicochemical features of odors contribute, at least in part, towards explaining the nature and origin of olfactory crossmodal correspondences.

## Supplementary Information

Supplementary Information 1.

Supplementary Information 2.
