# Supplementary material for: Physicochemical features partially explain olfactory crossmodal correspondences
Source: Sci Rep. 2023 Jun 30;13:10590. doi: 10.1038/s41598-023-37770-1 (PMC10313698; doi:10.1038/s41598-023-37770-1)
Supplement: Supplementary file 1 — Supplementary Information 1. [file 41598_2023_37770_MOESM1_ESM.docx]

**Physicochemical features partially explain olfactory crossmodal correspondences**

Ryan J. Ward^1,*^, Sophie M. Wuerger^2^, and Alan Marshall^1^

^1^University of Liverpool, Department of Electrical Engineering & Electronics, Liverpool, L69 3GJ, United Kingdom.

^2^University of Liverpool, Department of Psychology, Liverpool, L69 7ZA, United Kingdom.

* Corresponding Author

E-mail: [ryan.ward@liverpool.ac.uk](mailto:ryan.ward@liverpool.ac.uk)

**Table S1 | Generalized linear mixed model results for all perceptual dimensions.** The degrees of freedom for all coefficients in the table is 671 with the exemption of pitch model where the degrees of freedom are 591. Marginal *R*^2^ is the variance explained by the fixed factors and conditional *R*^2^ is the variance explained by the entire model (Nakagawa and Schielzeth, 2013). The Wilkinson model formula that was used is; Perceptual dimension ~ 1 + Air Quality + Temperature + Pressure + Humidity + Gas + MQ5 + MQ9 + HCHO + (1 | Participant ID) + (1 | Odor ID).

| Model | Model fit statistics  (Log-likelihood (LL), Akaike information criterion (AIC), Bayesian information criterion (BIC), Conditional *R*^2^ (C-*R*^2^), Marginal *R*^2^ (M-*R*^2^)) | | | | | Coefficients  Name (Standardised estimate, Estimate, SE, t-stat, p-value) |
| --- | --- | --- | --- | --- | --- | --- |
|  | LL | AIC | BIC | C-*R*^2^ | M-*R*^2^ |  |
| Angularity of shapes | 3064.6 | 3118.9 | -1520.3 | 0.18 | 0.18 | Air Quality (0.1141, 0.30, 0.25, 1.20, 0.23)  Temperature (-0.4315, -1.30, 0.22, -5.87, p < 0.0001)  Pressure (-0.0006, -0.002, 0.17, -0.01, 0.99)  Humidity (-0.2057, -0.56, 0.29, -1.91, 0.06)  Gas (-0.1852, -0.55, 0.24, -2.32, 0.02)  MQ5 (0.1188, 0.35, 0.24, 1.41, 0.16)  MQ9 (0.0049, 0.014, 0.27, 0.05, 0.96)  HCHO (0.0817, 0.25, 0.23, 1.08, 0.28) |
| Smoothness of texture | -1481.3 | 2986.5 | 3040.8 | 0.10 | 0.06 | Air Quality (-0.0174, -0.04, 0.23, -0.17, 0.86)  Temperature (0.1157, 0.31, 0.21, 1.5, 0.13)  Pressure (-0.0309, - 0.08, 0.15, -0.52, 0.60)  Humidity (-0.0083, -0.02, 0.270, -0.07, 0.94)  Gas (0.2416, 0.65, 0.22, 2.88, 0.0040804)  MQ5 (-0.1514, -0.39, 0.23, -1.72, 0.08)  MQ9 (-0.0468, -0.12, 0.25, -0.46, 0.64688)  HCHO (0.0897, 0.24, 0.22, 1.13, 0.26) |
| Perceived pleasantness | -1464.7 | 2953.4 | 3007.7 | 0.20 | 0.10 | Air Quality (0.2123, 0.50, 0.22, 2.26, 0.02)  Temperature (-0.0632, -0.17, 0.20, -0.87, 0.39)  Pressure (-0.1257, -0.33, 0.15, -2.26, 0.02)  Humidity (-0.0346, -0.08, 0.26, -0.32, 0.75)  Gas (0.4488, 1.21, 0.21, 5.67, p < 0.0001)  MQ5 (-0.5238, 1.37, 0.22, 6.29, p < 0.0001)  MQ9 (0.0434, 0.11, 0.24, 0.45, 0.65)  HCHO (0.3892, 1.07, 0.21, 5.19, < 0 .0001) |
| Pitch | -5940.9 | 11906 | 11958 | 0.36 | 0.09 | Air Quality (0.1027, 604.62, 526.36, 1.15, 591, 0.25)  Temperature (-0.1502, -1008.1, 464.84, -2.17, 0.03)  Pressure (-0.0506, -331.43, 346.18, -0.96, 0.34)  Humidity (0.0604, 362.68, 608.66, 0.60, 0.55)  Gas (0.0971, 650.94, 504.67, 1.29, 0.20)  MQ5 (-0.1142, -738.1, 511.84, -1.44, 0.15)  MQ9 (0.0954, 593.13, 569.5, 1.04, 0.30)  HCHO (0.2923, 1989.6, 485.74, 4.09, p < 0.0001) |
| L* | -2953.5 | 5931 | 5985.3 | 0.25 | 0.16 | Air Quality (0.3856, 8.43, 1.99, 4.23, p < 0.0001)  Temperature (0.0087, 0.21, 1.76, 0.12, 0.90)  Pressure (-0.2808, -6.83, 1.31, -5.21, p < 0.0001)  Humidity (0.2376, 5.29, 2.30, 2.30, p = 0.0218)  Gas (0.4562, 11.36, 1.91, 5.95, p < 0.0001)  MQ5 (-0.6921, -16.60, 1.94, -8.57, p < 0.0001)  MQ9 (0.3046, 7.03, 2.16, 3.26, p = 0.0012)  HCHO (0.5197, 13.13, 1.84, 7.14, p < 0.0001) |
| a* | -3248.6 | 6521.2 | 6575.5 | 0.22 | 0.17 | Air Quality (-0.1820 ¸ -6.12, 3.12, -1.96, 0.0503)  Temperature (0.3154, 12.09, 2.76, 4.39, p < 0.0001)  Pressure (0.0649, 2.43, 2.05, 1.18, 0.2378)  Humidity (0.4316, 14.79, 3.61, 4.10, p < 0.0001)  Gas (0.2671, 10.23, 2.99, 3.42, 0.0007)  MQ5 (0.0506, 1.87, 3.04, 0.61, 0.5388)  MQ9 (0.2255, 8.01, 3.38, 2.37, 0.0180)  HCHO (0.0869, 3.38, 2.88, 1.17, 0.24) |
| b* | -3339.7 | 6703.4 | 6757.6 | 0.25 | 0.23 | Air Quality (0.6529, 25.87, 3.61, 7.18, p < 0.0001)  Temperature (0.3255, 14.70, 3.18, 4.62, p < 0.0001)  Pressure (-0.072, -3.18, 2.37, -1.34, p = 0.18013)  Humidity (-0.1385, -5.59, 4.17, -1.34, p = 0.18016)  Gas (0.4794, 21.63, 3.46, 6.26, p < 0.0001)  MQ5 (-0.8851, -38.48, 3.50, -10.98, p < 0.0001)  MQ9 (-0.4705, -19.69, 3.90, -5.05, p < 0.0001)  HCHO (0.3337, 15.28, 3.33, 4.59, p < 0.0001) |
