# Supplementary material for: Physicochemical features partially explain olfactory crossmodal correspondences
Source: Sci Rep. 2023 Jun 30;13:10590. doi: 10.1038/s41598-023-37770-1 (PMC10313698; doi:10.1038/s41598-023-37770-1)
Supplement: Supplementary file 2 — Supplementary Information 2. [file 41598_2023_37770_MOESM2_ESM.docx]

**Supporting Information**

**Dataset S1** Raw perceptual and electronic nose data. The zipped directory contains all raw perceptual and physicochemical data in the form of as .csv files, and a read me file explaining its structure. Found at: doi:10.5281/zenodo.6166390
